# Factors Influencing Adaptation and Performance at Physical Exercise in Complex Congenital Heart Diseases after Surgical Repair

**DOI:** 10.1155/2014/862372

**Published:** 2014-04-15

**Authors:** P. P. Bassareo, L. Saba, P. Solla, C. Barbanti, A. R. Marras, G. Mercuro

**Affiliations:** ^1^Department of Medical Sciences “M. Aresu”, University of Cagliari, Policlinico Universitario, S.S. 554, Bivio di Sestu, Monserrato, 09042 Cagliari, Italy; ^2^Department of Imaging Sciences, University of Cagliari, Policlinico Universitario, S.S. 554, Bivio di Sestu, Monserrato, 09042 Cagliari, Italy; ^3^Department of Public Health, Clinical, and Molecular Medicine, University of Cagliari, Policlinico Universitario, S.S. 554, Bivio di Sestu, Monserrato, 09042 Cagliari, Italy; ^4^Department of Anesthesiology, Intensive Care, and Pediatric Cardiac Surgery, Necker Hospital for Sick Children, 149 rue de Sèvres, 75015 Paris, France

## Abstract

In the last thirty years, steady progress in the diagnostic tools and care of subjects affected by congenital heart diseases (CHD) has resulted in a significant increase in their survival to adulthood, even for those affected by complex CHD. Based on these premises, a number of teenagers and adults affected by corrected (surgically or through interventional techniques) CHD ask to be allowed to undertake sporting activities, both at a recreational and competitive level. The purpose of this review is to examine the mechanisms influencing the adaption at physical exercise of patients suffering from complex CHD. The conclusion is that even if there are some modest risks with exercise, they should be seen in perspective, and the life-long benefits of regular exercise on general health, mood, and well-being should be emphasized.

## 1. Introduction

Congenital heart diseases (CHD) represent a complex reality on a continuous upwards trend; their incidence is estimated between 6 and 8 per 1,000 live births. In addition, new diagnostic methods and modern surgical techniques have resulted in a survival rate to adulthood in approximately 80–85% of these patients [1, 2]. In this scenario, children, teenagers, and adults affected by* corrected *(surgically or through interventional techniques) CHD ask to be allowed to undertake sporting activities, both at a recreational and competitive level.

In this paper, CHD have been subdivided in an entirely arbitrary manner on the basis of a physiopathological criterion into the following groups:* simple *CHD and* complex *CHD.* Simple *CHD, which comprise the majority of diseases addressed by sports physicians, may be further divided into two subgroups:

1. those characterized by pulmonary hyperflow due to left-to-right shunt (such as interatrial septal defect and interventricular septal defect);
2. those characterized by left and right ventricular outlet obstruction (such as pulmonary and aortic stenosis).

*Complex CHD,* on the other hand, are represented by less easily identifiable defects, which are frequently accompanied by cyanosis due to the presence of a right-to-left shunt with subsequent mixing of oxygenated and deoxygenated blood.

CHD functional classification has been also reported in Table 1 [3].

Otherwise, CHD may be classified according to an anatomical criterion as well (Table 2).

Moreover, it should be taken into account that the majority of CHD have an underlying hereditary genetic basis and are often part of syndromic panels; associations with the malformation of other organs and systems should therefore be taken into consideration when assessing the suitability of a patient for recreational and competitive sporting activities (such as, e.g., Marfan's syndrome) [3, 4].

The purpose of this paper is to review the haemodynamic factors influencing both adaption and performance at physical exercise of patients suffering from complex CHD after surgery. In this respect, a Pubmed/Medline search was conducted using the MeSH terms:* congenital heart (cardiac) defect (disease), cardiac surgery, catheterization, tetralogy of Fallot, transposition of the great vessels, congenitally corrected of the great vessels, Ebstein's anomaly, univentricular heart, Fontan, physical exercise (performance), sport, cardiopulmonary test, exercise tolerance, cardiac rehabilitation, pulmonary arterial hypertension, sudden death, tachycardia, arrhythmia, bradycardia, atrioventricular block*,* psychology,* and their combinations. Articles identified in this manner were retrieved and their reference lists searched for additional relevant articles. The search was limited to English-language publications, but no other restrictions were applied. The Pubmed/Medline database was searched from its inception to September 2013. The most relevant articles have been reported.

## 2. Complex Heart Diseases and Physical Exercise

### 2.1. Tetralogy of Fallot

The tetralogy of Fallot (ToF) is the most common type of cyanotic CHD and is characterized by four main components:

1. subpulmonary infundibular stenosis;
2. defect of the subaortic interventricular septum due to misalignment;
3. aorta overriding the septum;
4. right ventricular hypertrophy [5].

Corrective surgical procedures are nowadays performed during the first year of life with significantly more positive results than those obtained in the past, when palliative interventions were carried out before reaching a complete correction.

However, progressive exercise intolerance is a common problem after ToF repair [6].

Many factors may influence the poor performance at physical exercise in operated ToF patients: residual right ventricular outflow tract obstruction; residual pulmonary insufficiency; impaired biventricular function; presence of ventricular or atrial arrhythmias [3].

A statistically significant difference has been previously demonstrated in VO_2 _peak between ToF subjects and healthy controls, irrespective of the surgical approach, age at surgery, and aortopulmonary shunts prior to performing total correction. This observation was irrespective of the apparent good clinical status of the subjects in the study as well [7].

Pulmonary regurgitation, which is usually more common and severe in ToF subjects after the use of a transannular patch during right ventricular outflow tract aggressive infundibulectomy and reconstruction, is probably the major determinant in influencing their physical performance. In fact, pulmonary regurgitation is responsible for progressive dilation of the right ventricle, arrhythmias, and sudden death in ToF individuals [8].

Several previous studies have reported an association between pulmonary regurgitation and exercise impairment, as testified by reduced exercise time, VO_2 _peak, and maximum achieved METs in comparison with healthy controls during exercise stress testing [9].

At short-term followup after surgery, this observation may be less evident or absent. Previous reports about this topic have been unable to demonstrate a relationship between the degree of pulmonary regurgitation and exercise capacity or right ventricular function few years after surgical repair [10]. This finding might be due to the different methods used to measure the degree of pulmonary regurgitation, and/or the cardiovascular compensation of severe pulmonary regurgitation with good chronotropic response to exercise. In this respect, performance at physical exercise may not significantly statistically differ from that of healthy peers [9].

Abnormal cardiac function and haemodynamic abnormalities secondary to pulmonary regurgitation and residual defects usually appear after longer periods of followup in ToF patients.

In fact, at mid and most of all long-term followup, the hemodynamic situation of ToF patients may be substantially different. As time passes, pulmonary regurgitation may lead to progressive right ventricle enlargement and dysfunction, with consequent biventricular dyssincrony, progression to heart failure, and poor performance at physical exercise [10–12].

Furthermore, ToF subjects with previous palliation by means of aortopulmonary shunts have shown lower exercise performance than those without previous aortopulmonary shunts. In fact, these shunts may induce changes in the pulmonary vascular system, which is pathologically altered and can, in turn, negatively affect exercise capacity [13].

Furthermore, ToF patients are at times characterized by a poor long-term survival rate, likely due to cardiac causes such as ventricular arrhythmias, with subsequent sudden death. A QRS prolongation ≥ 180 msec at basal electrocardiogram is a strong predictor for refining risk stratification for ventricular tachycardia in these patients [8].

QRS enlargement continues to confirm its efficiency in predicting increased risk for ventricular arrhythmia even when electrocardiogram has been performed during physical exercise.

Changes in QRS duration during exercise test have been calculated and compared with those of healthy controls. QRS duration was larger in ToF patients (*P* < 0.001) and correlated with right ventricle volume, right ventricle ejection fraction, right ventricle wall mass, pulmonary regurgitation percentage, and VO_2_ max.

In conclusion, during physical exercise inhomogeneity of repolarisation, known to predispose to reentry ventricular arrhythmia, increased in repaired TOF. A greater inhomogeneity was found in the presence of more severe pulmonary regurgitation [14].

Furthermore in adult patients with repaired ToF, QRS duration at rest seems to be a predictor of maximal exercise capacity, and changes in QRS duration are likewise associated with the latter. Thus, in a group of ToF patients, QRS duration in V1 lead (msec) was measured at rest, at maximal exercise (Wmax, Watt), and at peak oxygen consumption (peak VO_2_, mL/min). Stroke volume was calculated from cardiac output, obtained by CO_2_ rebreathing.

The study findings have shown that in patients with QRS shortening, peak VO_2_ and exercise-induced increase in stroke volume were significantly higher than in patients with QRS prolongation. This study indicated that QRS shortening during exercise in ToF patients was related to a better exercise performance [15].

On the other hand, postoperative exercise testing in patients with ToF can also unmask a complete heart block that was not elicited by the standard 12-lead electrocardiographic recording. Andersen and colleagues reported that 1% of ToF patients developed a complete heart block at long-term followup [16].

On the basis of the last Guidelines on eligibility in sport activities for GUCH, only ToF patients with normal or mild right ventricular outflow tract obstruction; no more than mild pulmonary insufficiency; normal or slightly impaired biventricular function; absence of ventricular or atrial arrhythmias may be involved in sport activities.

Specifically, in case of moderate residual lesion with right ventricular pressure less than 50% of systemic pressure, or residual ventricular septal defect, or moderate pulmonary regurgitation, but normal biventricular function, only* low static and dynamic sports *are allowed. A yearly followup is needed, with a complete reassessment every second year. Patients with conduit between right ventricle and pulmonary artery should avoid sports with risk of bodily collision [3, 17, 18].

### 2.2. Transposition of the Great Vessels

The transposition of the great vessels (D-TGV) is a CHD characterized by ventriculoarterial discordance: it means that the aorta, placed on the right side and at the front, originates from the right ventricle, whereas the pulmonary artery originates from the left ventricle. Two-thirds of cases are represented by* simple *forms, which are not associated with other defects.

In the past, surgical treatment was represented by a physiological but nonanatomical correction of the defect (atrial switch according to* Mustard and Senning procedures*, characterized by the repositioning of venous returns through an interatrial baffle).

The preferred surgical intervention is currently the arterial switch (*Jatene procedure*), in which the two vessels are repositioned correctly and the coronary arteries reimplanted. Such an intervention represents both a physiological and anatomical correction of the defect and is carried out during the first few days of life, obtaining significantly better results in terms of mortality rate and late morbidity.

When comparing the functional outcome and cardiorespiratory response to exercise in patients who have undergone arterial switch for D-TGV, with patients who have undergone atrial switch operation, and with normal controls, cardiorespiratory exercise function is at, or slightly below, the lower limit of normal in patients with arterial switch, while the lowest values were observed in those who had undergone atrial switch (aerobic capacity assessed by ventilatory anaerobic threshold: *P* < 0.001 patients versus healthy controls; aerobic exercise function assessed by the slope of oxygen uptake versus exercise intensity: *P* < 0.05 patients versus healthy controls; efficiency of the pulmonary gas exchange assessed by calculation of the slope of ventilation versus carbon dioxide output during exercise: *P* < 0.001 patients versus healthy controls) [19].

Even in asymptomatic D-TGV patients, exercise endurance and respiratory response appear generally altered after atrial switch operation [20].

In these patients, the morphological right ventricle serves as the systemic ventricle. This condition is frequently complicated by the development of right ventricular dilation, hypocontractility, failure, and tricuspid valve regurgitation and can explain the impaired peak exercise performance in these patients, which is mainly due to the inability to increase stroke volume and heart rate at higher exercise intensities. Senning repair and a well-preserved right ventricular function are related to a better VO_2_ peak. Furthermore, an active lifestyle has a positive effect on exercise capacity and perceived physical functioning. Therefore it might be indicated to encourage D-TGV patients to adopt a more physically active lifestyle [21].

On the other hand, early and intermediate-term results of the arterial switch operation for D-TGV are encouraging. However, some questions remain about the long-term outcome for these patients, who are at risk of reduced exercise capacity, with most reports focusing on chronotropic incompetence as the main cause [22].

Residual right ventricular outflow tract obstruction is relatively common after the arterial switch operation, but its effect on exercise capacity is unknown. In this respect, 60 D-TGV patients (44 males, age 13.3 ± 3.4 years) who had undergone a neonatal arterial switch operation were studied using the cardiopulmonary exercise test and transthoracic echocardiography. The authors have concluded that a reduced exercise capacity is relatively common in children and young adults who have undergone an arterial switch operation, but it does not decrease with age. The presence of residual right ventricular outflow tract obstruction seems to have a negative effect on their exercise capacity [23].

According to recent consensus documents, D-TGV patients corrected through Mustard and Senning procedures (atrial switch)* may not be allowed to perform any type of competitive sports*. However* recreational low to moderate dynamic and low static activities* are encouraged in carefully selected patients.

In patients corrected with arterial switch, the suitability for sporting activities should be assessed through physical examination (NYHA functional class), electrocardiogram, echocardiogram, chest X-ray, and exercise stress test. In patients with positive outcomes (i.e., those without or only mild neoaortic insufficiency, no significant pulmonary stenosis, no signs of ischaemia or arrhythmia on exercise electrocardiogram),* all sports are allowed, with exception of high static and high dynamic sports*. A yearly followup is recommended [3, 17, 18].

### 2.3. Congenitally Corrected Transposition of the Great Arteries

The congenitally corrected transposition of the great arteries (ccTGA) is a complex CHD characterized by a double atrioventricular and ventriculoarterial discordance: the left atrium is connected to a systemic morphologically right ventricle from which originates the aorta and the right atrium is connected to a morphologically left ventricle from which originates the pulmonary artery. The expression* congenitally corrected *refers to the haematic flow that follows a physiological course even if through anatomically transposed structures. Only 1% of cc-TGA cases present themselves as isolated defects, meaning that in almost all cases one or more associated defects may be observed [24].

Regardless of the latter aspect, two elements that negatively influence the prognosis of these patients can be detected: (1) the presence of a systemic morphologically right ventricle and atrioventricular valve (the right ventricle and the tricuspid valve, often dysplastic, are not designedto handle systemic pressure over long periods of time); (2) the location of the atrioventricular node, which very often influences the evolution towards complete atrioventricular block.

Although an isolated defect would not require surgical intervention, the constant association with other cardiac defects often calls for a surgical correction that varies from patient to patient [3].

The systemic morphologically right ventricle tends to respond differently to exercise in comparison to a normal morphologically left ventricle. It is known that the organization of the myocytes is different between the right and left ventricles. The left ventricular myocytes are arranged in layers of counter-wound helixes that surround the ventricular cavity, conferring a special twisting motion during systole and early diastole, and providing the optimal stress and strain to generate the necessary forces to sustain the demand on a systemic ventricle [25]. The morphologically right ventricle lacks the helical myocytic arrangement, lacking also the twisting or torsion component conferred by the helical arrangement, and thus being unable to sustain the demands of a systemic ventricle. When in systemic position, the morphologically right ventricle is also unable to respond to an increasing work demand, such as during exercise, in the fashion of the normal systemic ventricle [26].

Systemic ventricular dysfunction is also caused by volume overload due to atrioventricular valvar regurgitation or abnormal myocardial perfusion, with ischemia of the myocardium during periods of increasing demand underscoring the abnormal myocardial function [27–29].

Few studies have been reported in literature about the performance at physical of ccTGA patients. In one of these reports, the finding has shown diminished values of heart rate, forced expiratory volume in one second (FEV_1_), forced vital capacity, and systolic blood pressure compared to the predicted values. These poor results may contribute to the reduced maximal oxygen uptake (VO_2 _peak) found in patients with ccTGA. In addition, a limited increase in systolic right ventricular ejection fraction and a decrease in pulmonary left ventricle contractility have been found, thus suggesting a dysfunction of both ventricles [30].

Cc-TGA patients may not be allowed to perform any competitive sporting activities.

Patients may be allowed to take part in* recreational low to moderate dynamic and low static activities*, only following a personalized evaluation performed by highly trained experts [17, 18].

### 2.4. Ebstein's Anomaly

Ebstein's anomaly (EA) is a rare CHD characterized by a wide spectrum of anomalies of the tricuspid valve and right ventricle. Its main feature is a displacement of the septal leaflet of the tricuspid valve downwards the apex of the right ventricle with subsequent formation of two cardiac chambers: (1) the first one, supravalvular, being formed by the right atrium and a part of the “atrialized” right ventricle; (2) the second one consisting in the remaining portion of the subvalvular right ventricle.

The annulus of the tricuspid valve is still in the normal position. EA is frequently associated with a number of other heart defects (e.g., atrial septal defect), and arrhythmias due to the presence of accessory beams.

The functional impairment of the right ventricle and regurgitation of the tricuspid valve retard forward flow of blood through the right side of the heart. In addition, during contraction of the atrium, the “atrialized” portion of the right ventricle balloons out and acts as a passive reservoir, decreasing the volume of ejected blood. The overall effect on the right atrium is dilatation, thus increasing the size of the interatrial communication. Tricuspid regurgitation increases by annular dilatation. Associated heart diseases in EA have a further effect on EA pathophysiology [31].

This pathophysiological spectrum results in lower VO_2 _peak, and higher VE/VCO_2_ slope in comparison with healthy peers. In addition, the exercise function of patients with EA tends to deteriorate over time. This deterioration appears to be related to a progressive decline in their ability to augment their forward stroke volume and heart rate during exercise. Exercise capacity in patients with EA becomes gradually lower alongside the grade of the lesion severity [32–34].

Surgical intervention is indicated for the following conditions:

- limited exercise capacity (NYHA III-IV),
- increasing heart size (cardiothoracic ratio greater than 65%),
- important cyanosis (resting oxygen saturation of less than 90%. Level B),
- severe tricuspid regurgitation with symptoms,
- transient ischemic attack or stroke [35].

Patients who require operation for EA should be operated only by CHD surgeons who have substantial specific experience and success with this operation. Every effort should be made to preserve the native tricuspid valve [35].

Recently, in 21 patients with EA (age range: 6–59 years; 16 female, 5 male) who underwent surgery for tricuspid regurgitation and, if present, closure of an interatrial shunt, a cardiopulmonary exercise test has been performed prior to and 6 to 18 months after surgery. After surgery, peak oxygen uptake ameliorated from 68.4% to 77.3% of the predicted value (*P* = 0.009). Ventilatory efficiency (VE/VCO_2_ slope) improved from 32.5 to 29.3 (*P* = 0.001). In 14 patients with additional interatrial shunt closure, oxygen saturation improved from 95% to 99% at rest (*P* = 0.003) and from 88% to 99% under peak exercise (*P* = 0.003). Improvements in VE/VCO_2_ slope were similar in EA patients who had undergone primary surgery (*P* = 0.005) or reoperation (*P* = 0.018). There was no difference between tricuspid valve repair and replacement in the short-term followup [36].

Even at long-term followup after repair of EA, exercise tolerance appeared improved (70 ± 19% to 92 ± 9% of predicted values, *P* < 0.05). Both tricuspid function and NYHA functional class have remained essentially unchanged at the end of followup, indicating good haemodynamic and functional results [37].

According to Guidelines, subjects affected by surgically treated or not EA* may not take part in competitive sports*. Patients may be allowed to take part in* recreational low to moderate dynamic and low static activities* [17, 18].

## 3. Complex CHD Surgically Corrected by Fontan Repair 

The present subparagraph refers to a group of particularly complex and rather heterogeneous CHD (tricuspid or mitral valve atresia, double inlet left ventricle, univentricular heart, hypoplastic left or right ventricle and heterotaxy syndromes) that share the impossibility to be subjected to biventricular correction.

A heart corrected with a Fontan repair (UVH-F) is a CHD in which a single pumping chamber is responsible for both systemic and pulmonary circulations. The UVH-F consists of diverting systemic venous blood directly to the pulmonary circulation without the presence of a prepulmonary pump to add forward energy to flow through the lungs. Flow return from the pulmonary vascular bed to the single ventricle is thereby markedly reduced, thus resulting in a decreased/absent preload reserve. In this respect, cardiac output at rest in UVH-F decreases to 50–80% of the normal value. The preload insufficiency is made more apparent by a dilated, hypertrophic, and asynchronously hypocontractile single ventricle [38, 39].

It is well documented that children with a Fontan circulation have a reduced exercise capacity. They have a unique physiological response to exercise, testified by a reduced VO_2 _peak, compared with healthy controls [40].

The reduced VO_2 _peak can be caused by a combination of several factors, such as chronotropic incompetence [40, 41]. Moreover, a reduced cardiac stroke volume might also be a limiting factor for these patients [38, 42]. It might be explained by a limited diastolic return to the systemic ventricle, or by a chronic volume overload, or diminished contractile response and impaired diastolic filling [43–45].

At rest and up to submaximal levels of exercise, arterial blood pressure is relatively well-maintained in Fontan patients, partly due to increased systemic arterial resistance [38]. Peak blood pressure, however, is usually lower than in healthy subjects due to an impaired increase in cardiac output at peak exercise [46].

Peak minute ventilation (VE) during exercise is decreased in UVH-F patients. It might be explained by a low nonpulsatile pulmonary blood flow responsible for a deterioration in gas exchange in the lungs [47, 48]. Moreover, an increased pulmonary vascular resistance, which is frequently observed in several CHD, might contribute to an impaired gas exchange [49].

Moreover, patients with a Fontan circulation have small lungs, requiring a higher breathing frequency to maintain a sufficient ventilation, however, reduced, if compared with healthy subjects [47, 48].

Ventilation/perfusion mismatching in UVH-F patients, with subsequent reduction in SaO_2_%, is frequently observed during exercise, showing the reduced arterial oxygen saturation and hence limitations in oxygen transport [40]. A reduced SaO_2_% contributes further to a limitation in VO_2 _peak [38].

Moreover, previous reports have found a muscle-skeletal impairment in Fontan patients, as testified by reduced muscle blood flow during exercise in children, slower oxygen uptake kinetics, which reflects the impaired oxygen delivery at their working muscles, and other intrinsic muscle metabolic abnormalities [50–53]. Moreover, if compared with healthy peers, a reduced body mass is frequently observed in these patients. A lower muscle mass also contributes to a lower oxygen utilization during exercise and a lower VO_2 _peak [54].

Based on these premises, UVH-F patients appear to be better adapted to perform submaximal physical exercise, which in any case is not different to that of their healthy peers [55].

According to the last European Guidelines, UVH-F patients who are in good hemodynamic condition are allowed to take part in recreational activities from low to moderate and low static group [17, 18]. However, in another study the combination of strength and aerobic exercises was advocated [56]. All the types of competitive sporting activities are not allowed in these subjects [17, 18].

It has been demonstrated that, after following an exercise training programme, these patients have resulted in an improved exercise capacity. Specifically, taking into account a number of studies performed in this field, an improvement in VO_2 _peak ranging from 0% to 22% has been demonstrated. Furthermore, improvements in walking distance, respiratory muscle oxygenation, and blood pressure ergoreflex were registered [42].

However, these results were related to Fontan patients in good hemodynamic conditions. Usually, those with ECG alterations, arrhythmias, reduced pumping function of the heart, and systemic desaturation (<80%) are not eligible for physical exercise [57, 58]. The presence of clinical relevant arrhythmias is maybe the major contraindication for physical exercise in these patients [59].

Fontan completion at a younger age is associated with better exercise performance in adolescents [60].

However, more researches are needed to establish the optimal exercise mode, and the effects of exercise training on cardiac and peripheral muscle function, physical activity, and health-related quality of life in UVH-F individuals.

At this time, the only certainty is that—according to the last Guidelines—among UVH-F patients only those asymptomatic (class NYHA II), with absence of residues or enlargement of the right atrium varying from moderate (classic Fontan) to absent (total cavopulmonary anastomosis), ejection fraction of the systemic ventricle 40–50%, absent or light atrioventricular valvular insufficiency, effort tolerance and/or physical efficiency 70–80% of normal standards (VO_2_ max > 25 mL/kg/min), absent or moderate systemic desaturation (85–90%), absence of arrhythmia or supraventricular extrasystole, episodes of supraventricular tachycardia controlled by medical therapy and/or radio frequency ablation procedure, and possible normofunctional pacemaker, may be allowed to take part in recreational activities from low to moderate and low static group [17, 18].

In recent years, new treatment strategies have been developed to improve exercise tolerance in UVH-F subjects. Due to the lack of a subpulmonary pumping ventricle, their cardiac output differs from normal and becomes quite complex, depending on several factors: preload, contractility, heart rate, pulmonary vascular resistance (which is the major determinant of cardiac output), and afterload [38].

This provides a potential explanation as to why pulmonary vasodilators may improve exercise tolerance. As it has been recently demonstrated, these agents (such as bosentan, sildenafil, iloprost) may offer an important new treatment strategy to partially overcome the physiological limitations in the physical performance at exercise of UVH-F patients [61, 62].

## 4. Conclusions

In conclusion, the steady progress in diagnostic methods and modern surgical techniques has resulted in an increasingly higher survival rate to adulthood of the majority of patients suffering from CHD [1, 2]. In this scenario children, teenagers, and adults affected by corrected (surgically or through interventional techniques) CHD ask to be allowed to undertake sporting activities, both at a recreational and competitive level.

Consensus reports have stated that exercise should be encouraged and regularly performed in these patients, but this is not still common practice. Instead, it should be taken into account that participation in a physical exercise training program is safe and improves fitness in children and young adults with CHD [63]. While there are some modest risks with exercise, they should be seen in perspective, and the life-long benefits of regular exercise on general health, mood, and well-being should be emphasized [64]. In addition, in CHD patients physical deconditioning and inactivity may further impair their hemodynamic status, thus increasing their risk of morbidity and mortality as well. If possible, since hospital discharge they should be included in a formal cardiac rehabilitation program, able to ameliorate their exercise performance.

In complex CHD, this improvement is mediated most of all by an increase in stroke volume and/or oxygen extraction during exercise.

Routine use of formal cardiac rehabilitation may greatly reduce the morbidity of complex CHD subjects [57].

In these patients, even depressive symptoms, low self-esteem, and quality of life, which are frequently associated with initial reduced exercise capacity and physical activity, may be improved by physical training [65].

## Figures and Tables

**Table 1:** Pathophysiological classification of congenital heart diseases (CHD).

	(1) *Simple CHD *
	(a) CHD characterized by pulmonary hyperflow due to left-to-right shunt (e.g., atrial septal defect and ventricular septal defect)
	(b) CHD characterized by left and right ventricular outlet (e.g., aortic valve stenosis and pulmonary valve stenosis)
	(2) *Complex CHD* (tetralogy of Fallot, transposition of the great vessels, congenitally corrected transposition of the great vessels, Ebstein's anomaly, complex CHD corrected according to Fontan procedure)

**Table 2:** Anatomical classification of congenital heart diseases (CHD).

	(1) *Shunt lesions* (atrial septal defect, ventricular septal defect, complete atrioventricular canal defects, patent ductus arteriosus, total anomalous pulmonary venous connection, partial anomalous pulmonary venous connection, aortopulmonary window, anomalous left coronary artery)
	(2) *Obstructive lesions* (Ebstein's anomaly of the tricuspid valve, tricuspid atresia, hypoplastic right ventricle ranging from critical pulmonary stenosis to pulmonary atresia with intact ventricular septum, double-chambered right ventricle, valvar pulmonary stenosis, congenital mitral obstruction, cor triatriatum, hypoplastic left heart syndrome ranging from mitral to aortic atresia, fixed left ventricle outflow obstruction, hypertrophic cardiomyopathy, valvar aortic stenosis, coarctation of the aorta, interrupted aortic arch)
	(3) *Conotruncal abnormalities* (tetralogy of Fallot, absent pulmonary valve syndrome, simple or D-transposition of the great vessels, congenitally corrected or L-transposition of the great vessels, double outlet right ventricle, truncus arteriosus)
	(4) *Complex heart diseases* (heterotaxy syndrome, single ventricle, straddling atrioventricular valves, juxtaposition of the atrial appendages, cardiac malpositions)

## Acronyms

CHD: Congenital heart diseases
ToF: Tetralogy of Fallot
D-TGV: Transposition of the great vessels
ccTGA: Congenitally corrected transposition of the great vessels
EA: Ebstein's anomaly
UVH-F: Univentricular heart corrected according to Fontan repair
VE: Ventilatory efficiency
VE/VCO_2_: Ventilatory equivalent ratio for oxygen and carbon dioxide
SaO_2_: Oxygen saturation
CO_2_: Carbon dioxide.
